# Supplementary material for: Multi-omic analysis of SDHB-deficient pheochromocytomas and paragangliomas identifies metastasis and treatment-related molecular profiles
Source: Nat Commun. 2025 Mar 17;16:2632. doi: 10.1038/s41467-025-57595-y (PMC11914184; doi:10.1038/s41467-025-57595-y)
Supplement: Supplementary file 1 — Supplementary Information [file 41467_2025_57595_MOESM1_ESM.pdf]

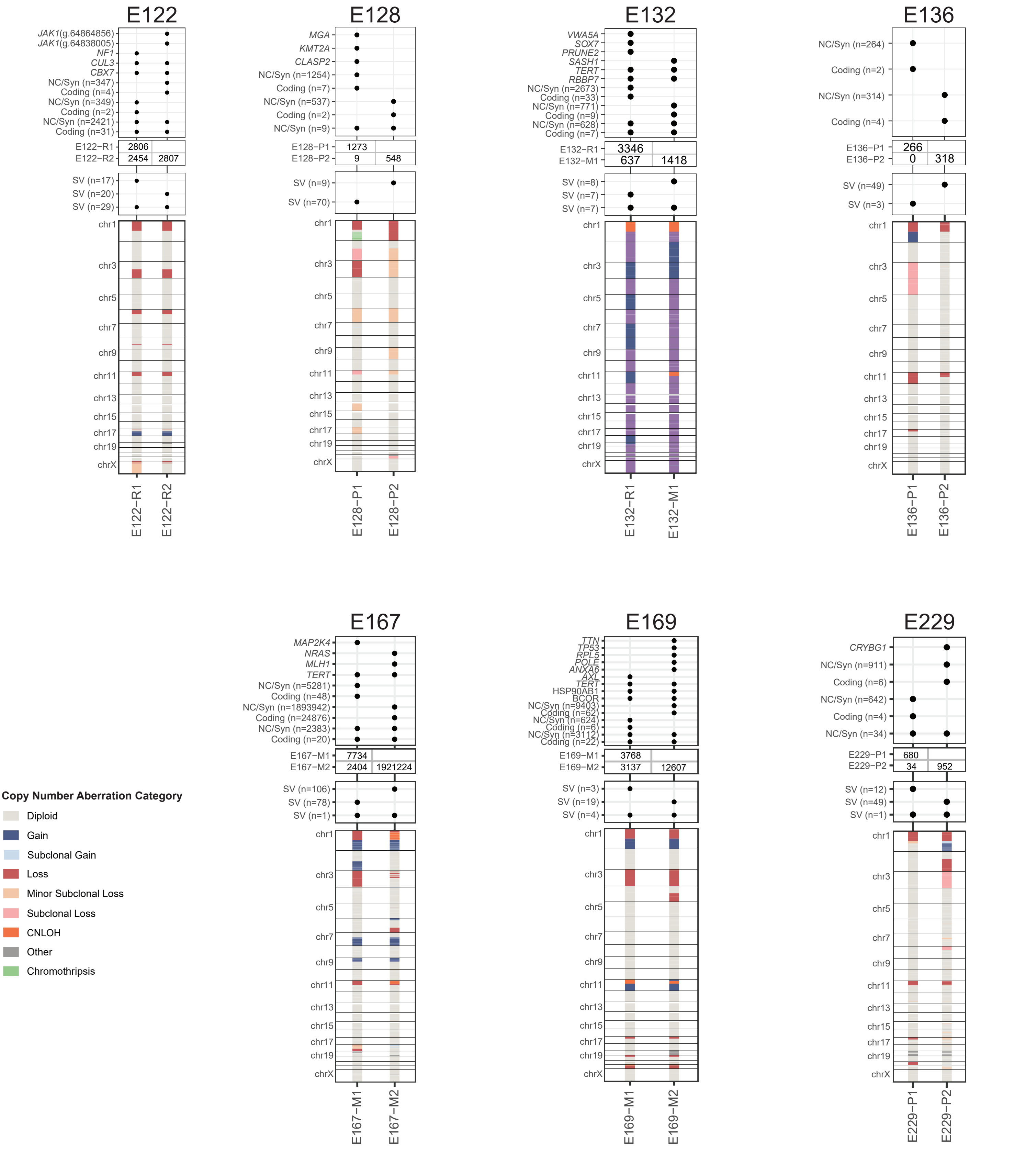

Supplementary Figure 1 Concordance of somatic alterations in paired tumours from individual patients
Single nucleotide, insertion/deletion, and structural variant-calling, as well as copy number alteration profiling was performed on multiple tumour samples originating from the same patient. The top panel of each plot shows the presence or absence of specific gene mutations or summarised mutation classes based on mutation consequence, where the total number of variants in each class is shown in parenthesis. The second panel from the top shows the number of shared variants when comparing the sample marked on the x-axis with that marked on the y-axis, when comparing a sample with itself the number represents the total mutation count for that sample. The second panel from the bottom shows the presence or absence of observed structural variants grouped by their status as private or shared, the total variants in each privacy class is shown in parentheses. The bottom panel shows the copy number alterations observed in each sample with line colour indicating the type of copy number event.

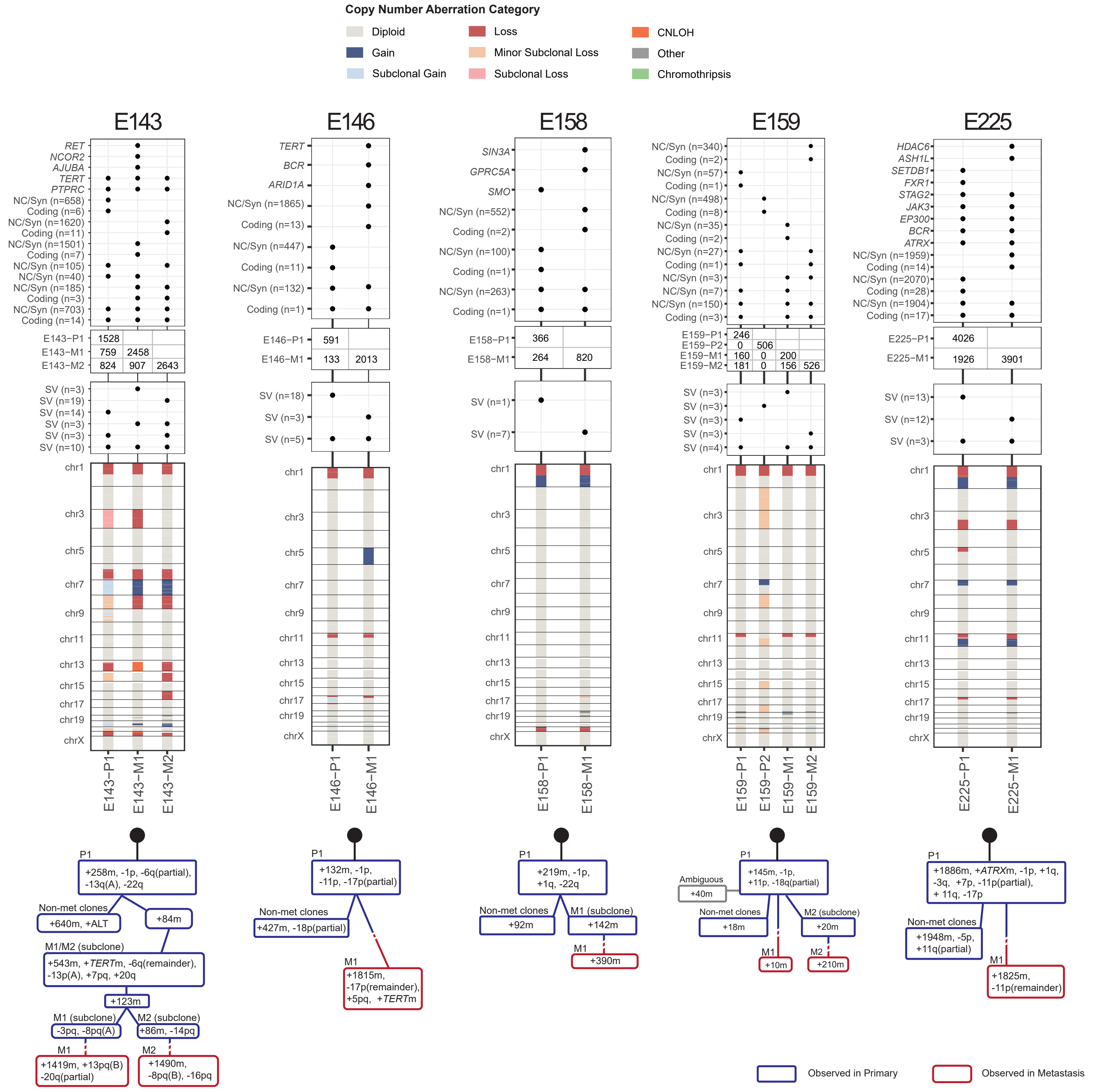

Supplementary Figure 2 Clonal evolution in paired primary and metastatic tumours
Single nucleotide, insertion/deletion, and structural variant-calling, as well as copy number alteration profiling was performed on multiple tumour samples originating from the same patient. The top panel of each plot shows the presence or absence of specific gene mutations or summarised mutation classes based on mutation consequence, where the total number of variants in each class is shown in parenthesis. The second panel from the top shows the number of shared variants when comparing the sample marked on the x-axis with that marked on the y-axis, when comparing a sample with itself the number represents the total mutation count for that sample. The third panel from the top shows the presence or absence of observed structural variants grouped by their status as private or shared, the total variants in each privacy class is shown in parentheses. The second panel from the bottom shows the copy number alterations observed in each sample with line colour indicating the type of copy number event. The bottom section shows a reconstruction of the clonal evolution of each tumour based on small mutations and copy number events. Mutations and copy number events observed in the primary sample are shown in blue boxes and those private to metastatic samples are shown in red boxes.

Ai

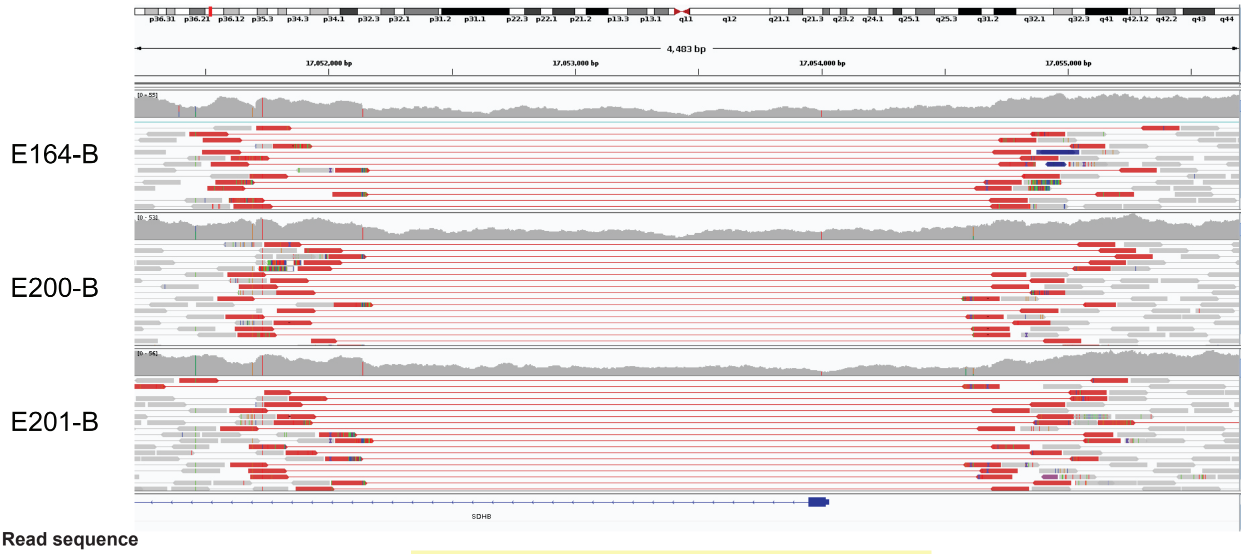

ii

Read sequence

CGGGCTGGTCTTGAACTCCTGACCTCAGGTGATCCACCCACCTTGGCCTCCCAAAGTGCTGGAATTACAGGCATGAGCCACTGCGCCCGGCCAATTTTTTTTTTTTTTT

Genomic sequence

ATTGGCAACCTTATCAGTCTTTGTATTATTCTGATTTGTAAATAGGTATCCTAGCCCTGCCAGCCTAAGGAACAAGATGCACATTTTTTAAATTTTTTTTTTTTTTTTG
AGACTGAGTTTCGCTCTTGTCGCCCAGGATGGAGTGCAATGGTGCGATTTCAGCTCACTGCACCCTCCACCTCCCAGGTTCAAGCAATTCTCCTGCCTCAGCCTCCTGT
GTAGCTAGGATTATAGGCGCCCACCACCACGCCCAGCTAATTTTTTTTTTATTTTTTTAGTAGAGACGGGGTTTCACCATGTTGGCCGGGCTGGTCTTGAACTCCTGAC
CTCAGGTGATCCACCCACCTTGGCCTCCCAAAGTGCTGGAATTACAGGCATGAGCCACTGCACCTGGCAAGATACACACTTTTAAGTAGGTAGTTCTTGTCCTAAAGGA
GTTCATAGTCCAGATGAAGAAATAAGGCAACACAAACAATAGTTAATATACTACAGTCAAATACTTACAGGCAGAATTGTGGTTCAGTGGTCAGACTATAAGCATAAAT
[..2109bp..]
TTATTCCTGGAATTTTTGTTTGTTTGTTTGAGATGGAGTCTCGCACTGTTTCCCGGGCTGGAGTGCAATGGCGCGATCTTGGCTCACTGCAACCTCCGCCTCCCGGGTT
CACGCGATTCTCCTGCCTCAGCCTCCCGAGTAGCTGGGATTACAGGCGCACACCATCACACCCGGCTAATTTTTTGTATTTTTAGTAGAGACGGGGTTTCATCATCTTG
GCCAGACTGGTCTTGAACTCCTGACCTCGTGATCCGCCCACCTTGGCCTCCCAAAGTGCTGGAATTACAGGCATGAGCCACTGCGCCCGGCCAATTTTTTTTTTTTTTT
TCAGACAGGGTCTCACTCTGTTGCCCAGGCCGGTGTGCAGTCTTGGCTCACTGCAGCCTCGACTTCCCAGGCTCAAGCGATCCTCCCACCTCCAGAGTAGCTGGGACTA
CAGGCGTGTGAGCCACCATACCCGGGTAGTTTTTTTTTTTTTCTTTTTTTTGAGATGGAGTATCGCTCTGTCTCCCAGGCTGGAGTGCAGTGGCGCGATCTCGGCTGAC

Bi

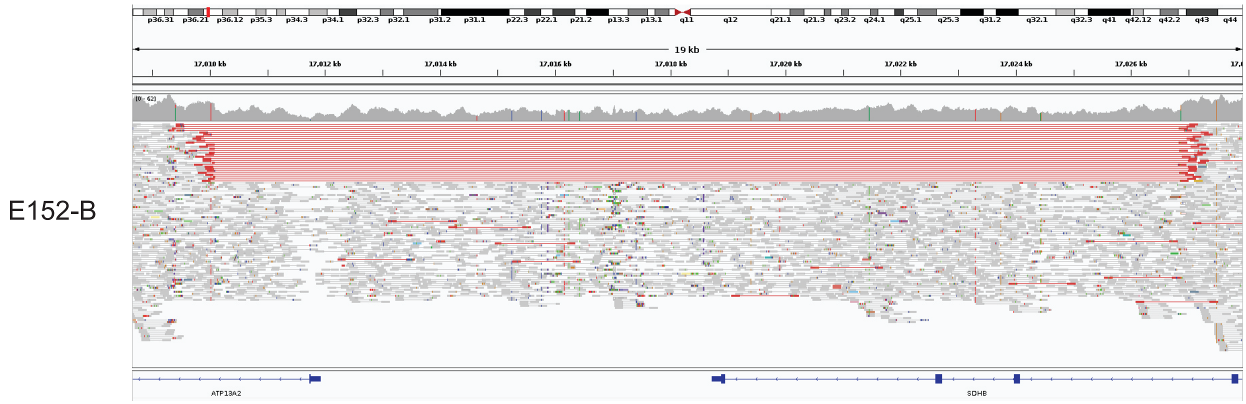

ii

Read sequence

TGCACCCTCCACCTCCCAGGTTCAAGCAATTCTCCTGCCTCAGCCTCCTGAGTAGCTGGGACTATCAGAATGCACCACTGCATCTGGCCTTTTAAAAAATTTATATATA

Genomic sequence

ACCAGCCCAGAACAGGCACCCACAGGCTGTGTGGGGGGGCGAGAGCACCTTCATGATGGCATTGCTCTTTTTTTTGAGATGGAGTCTTCCCCCCCCGTTCCCCCCTTCC
CCCCTGCCCGTCTCCCAGGCTGGAGTGCAGTGGAGAGATCTCAGCTCACTGCACCCTCCACCTCCCAGGTTCAAGCAATTCTCCTGCCTCAGTTCCTGAAGTAGCTGAG
ACTACAGGCACATGCCACCACACTCAGCTAATTTTTGTATTTTTAGTAGAGACGGGGTTTCCCCATGTTGGCCAGACTGGTCTTGAACTCCTGACCTCAAGTAATCCGC
CTGCCTCAGGCTCCCAAAGTGCTGGGATTACAGGCGTGAGCCACCACGCCTGGCCTGTCATTGCTCTTCTGATGAACAGCAGCTCCCATGGAGTGCTCACCATGCCCTG
CGGAAGCCCTTCAGTGTGCTAACTCAATGAAGCTTCACACACAACCCTCTGGGGTAGTGACTATTATTATCCCCATTGTACACCTGGGGAAACTGAGGAACAGCATGTG
[...16119bp...]
ATTACAGATGAGCCACTGCACCTGGCCGGACTCCCCTTTCTTAACGTTTTATGGGCAACAGGAGAGGATGCTAAATATTTACCAAGTCTCAAAAGGTAGAATTAATTTA
TTCATTCATACAACTGATACTTTTCATATATTCACATATATGTATATGTTTTAGAGACAAAACCTGGGCTATGTTGCCCGGGCTGGCCTCAAACTCTCAGACTCTCAAA
CTCCTGGGCTCAGGTGATCCTCCTACCTCAGCCTCCTGAGTAGCTGGGACTATCAGAATGCACCACTGCATCTGGCCTTTTAAAAAATTTATATATATTTTTACAGTAT
TTACATAACTAATATTTATGGATAAAATAACATGCGCCAGGGCCTAGGGATCCAGAAAGGAAGAAGACACAGCCATTGCCCTTGGCCCTGTGCAGACCTGCAGGTGGGG

Supplementary Figure 3 Homologous sequence surrounding breakpoints in germline SDHB structural variants

Structural variant calling was performed on whole genome sequencing data from germline DNA. **(Ai)** Integrated Genomics Viewer snapshot shows alignment of paired reads with larger than expected insert size and concordant drop in read coverage indicating a deletion event. Data is shown from three unrelated patients with a shared breakpoint. **(Aii)** The sequence surrounding the breakpoints is shown. Sequence upstream of the breakpoint is shown in blue, sequence (abridged) lost due to the deletion is shown in red, and sequence downstream of the breakpoint is shown in green. Where sequence homology was present around the breakpoint a yellow highlight has been applied. **(B)** Breakpoint for patient E152. Figure interpreted as per panel A.

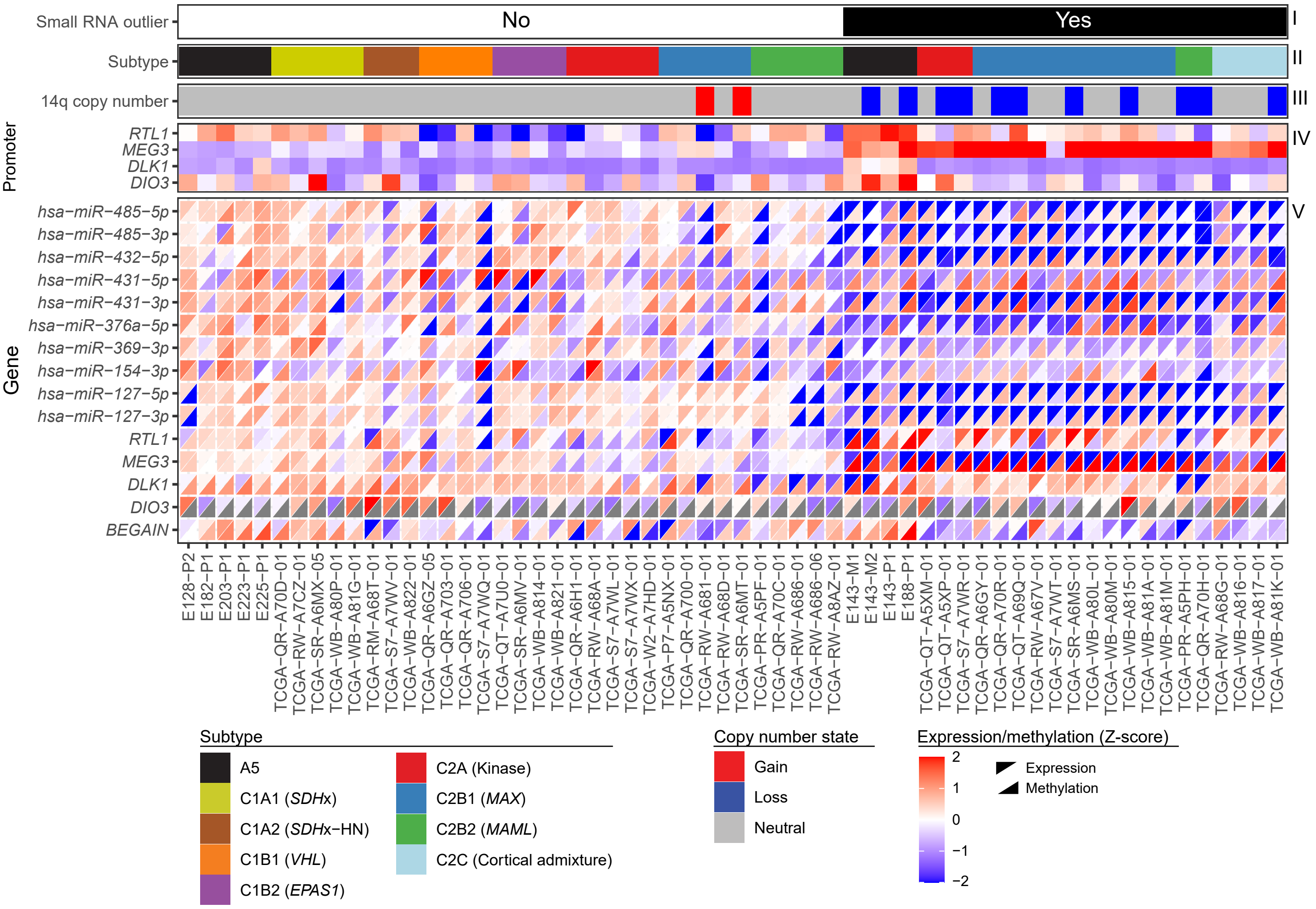

Supplementary Figure 4 Expression of genes in the imprinted regions of chromosome 14
(I) Membership in the outlier group identified by UMAP clustering of small-RNA sequencing. All samples in the outlier group and a random sampling of samples from each PCPG subtype (indicated in **II**) are shown. **(III)** Copy number status of chromosome 14q **(IV)** Average methylation of promoter associated probes for several imprinted genes are shown as a M-value Z-scores. **(V)** Expression (top-left triangle, log2-CPM Z-scores) and gene-body methylation (bottom-right triangle, M-value Z-scores) of genes and miRs within the chromosome 14 imprinted region.

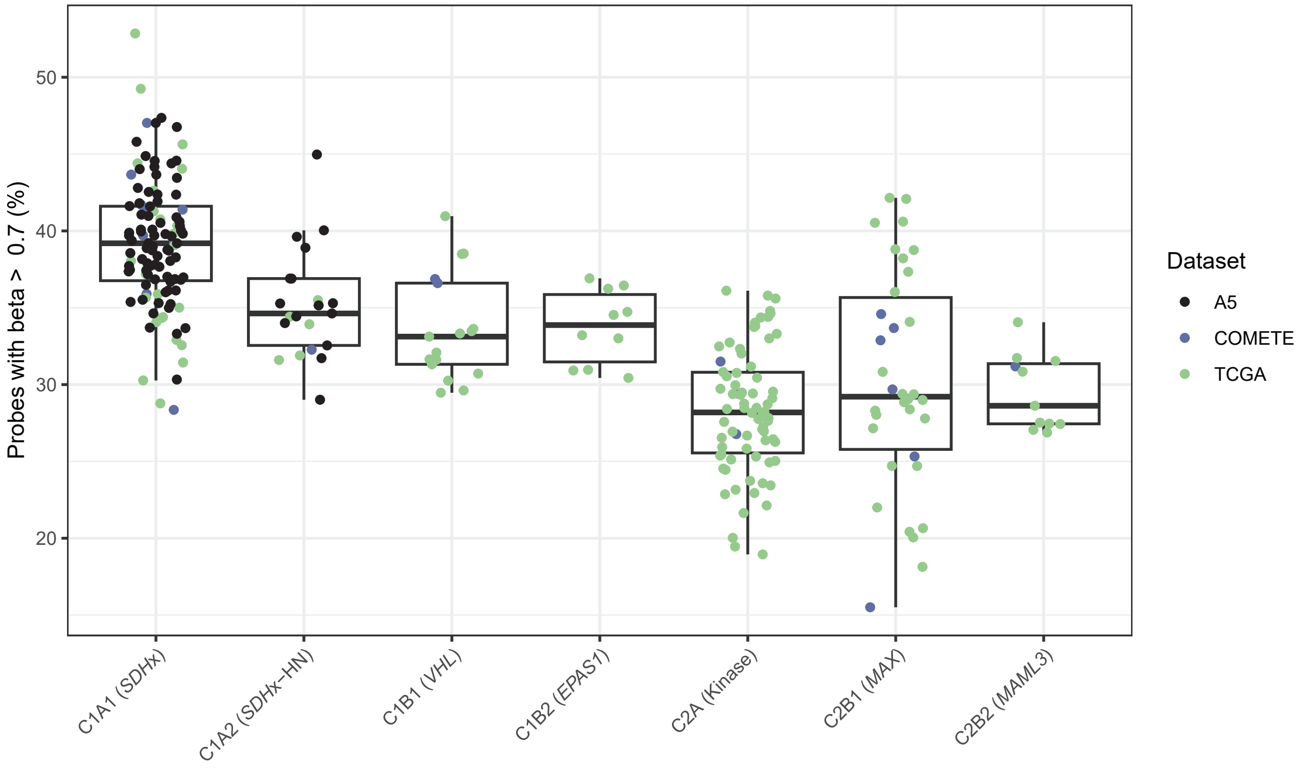

Supplementary Figure 5 Percentage of probes methylated
Array based methylation profiling data from the TCGA and COMETE studies was combined with the A5 dataset and subset to probes present on the Illumina 450K array format. For each sample (n=413), the proportion of all probes with a beta-value greater than 0.7 was tallied (y-axis). Samples are stratified by PCPG subtype (x-axis) and coloured by the source dataset. The lower and upper hinges of each boxplot correspond to the first and third quartiles, respectively, and the median value is marked. The whiskers extend to the largest and smallest value no greater than 1.5 times the interquartile range above or below the upper and lower hinges, respectively.

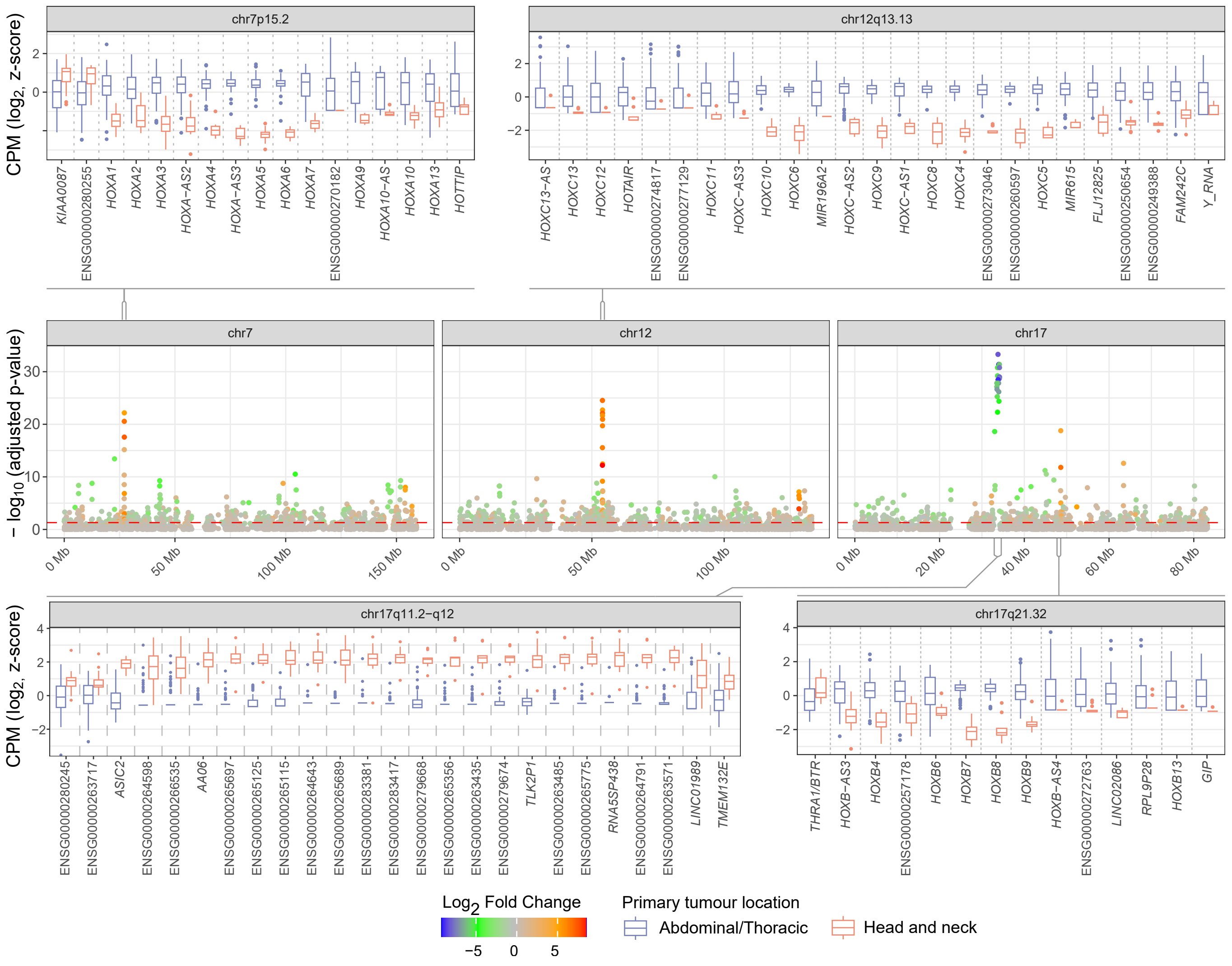

Supplementary Figure 6 Regional enrichment of genes differentially expressed between parasympathetic (non-chromaffin) HN-PG and sympathetic (chromaffin) PCPG
(Middle row) P-values adjusted for multiple testing (y-axis, -Log10) for each gene along chr7, chr12, and chr17 by gene-start chromosomal coordinate (x-axis). Colouring indicates the fold-change (log2). **(Upper/Lower rows)** Box plots showing the expression (y-axis, log2 CPM, z-score) of genes (x-axis) found in regions enriched for differentially expressed genes. Box-plot colour indicates contrast group (n = {PCPG: 75, HN-PG:12}). . The lower and upper hinges of each boxplot correspond to the first and third quartiles, respectively, and the median value is marked. The whiskers extend to the largest and smallest value no greater than 1.5 times the interquartile range above or below the upper and lower hinges, respectively. Values beyond the whisker extents are deemed outliers and are plotted individually.

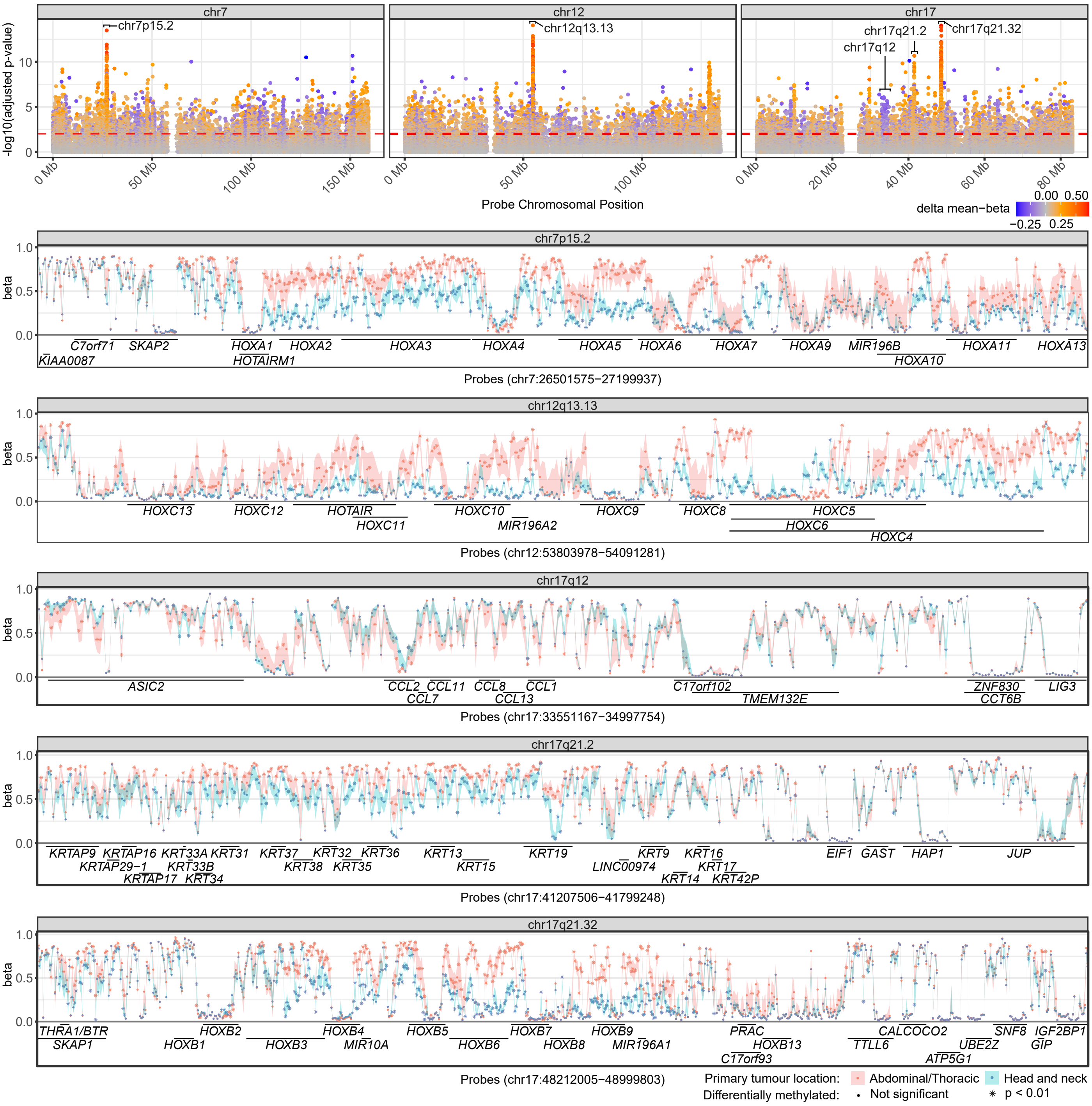

Supplementary Figure 7 Regional enrichment of probes differentially methylated between head and neck PGL and abdominal-thoracic PCPG

(Top row) P-values adjusted for multiple testing (y-axis, -Log10) for each probe along chr7, chr12, and chr17 by probe chromosomal coordinate (x-axis). Colouring indicates the difference in the average beta-value between contrast groups. **(Lower rows)** Median (points), 25% percentile (lower shaded region bounds), and 75th percentile (upper shaded region bounds) of beta-values (y-axis) for each probe within each contrast group (colour) along the respective chromosomal region (x-axis).

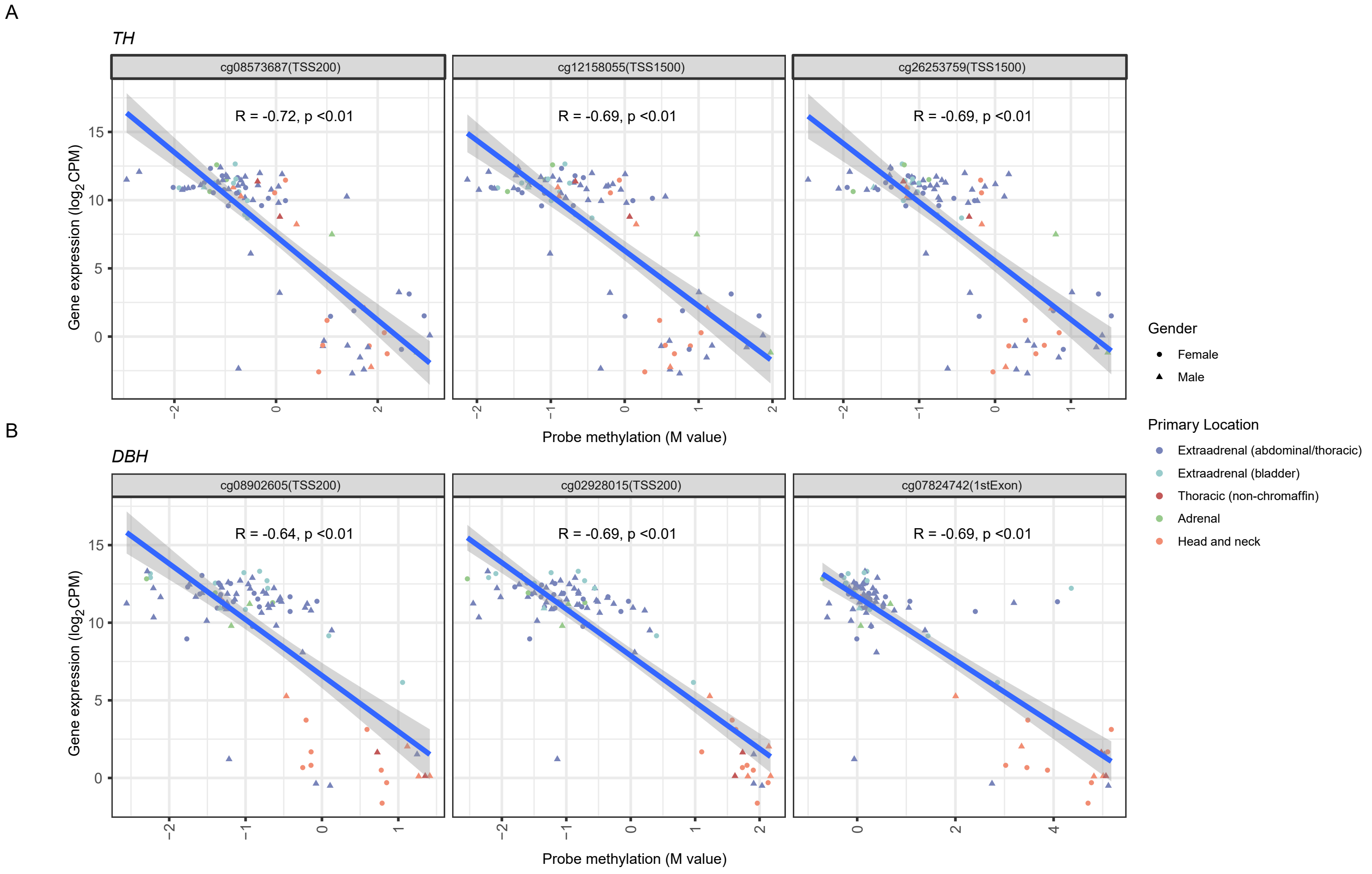

Supplementary Figure 8 Methylation proximal to the gene transcription start site correlates with gene expression for *TH* and *DBH*
Methylation (x-axis, M-values) of probes located 200 bp (TSS200) or 1500 bp (TSS1500) upstream of the transcription start site, or within the first exon (1st Exon) versus gene expression (y-axis, log2 CPM) of (A) *TH* or (B) *DBH*. A linear regression line (blue line), 95% confidence interval (shaded grey area), and correlation coefficient (R) for a Spearman correlation are provided (n = 88).

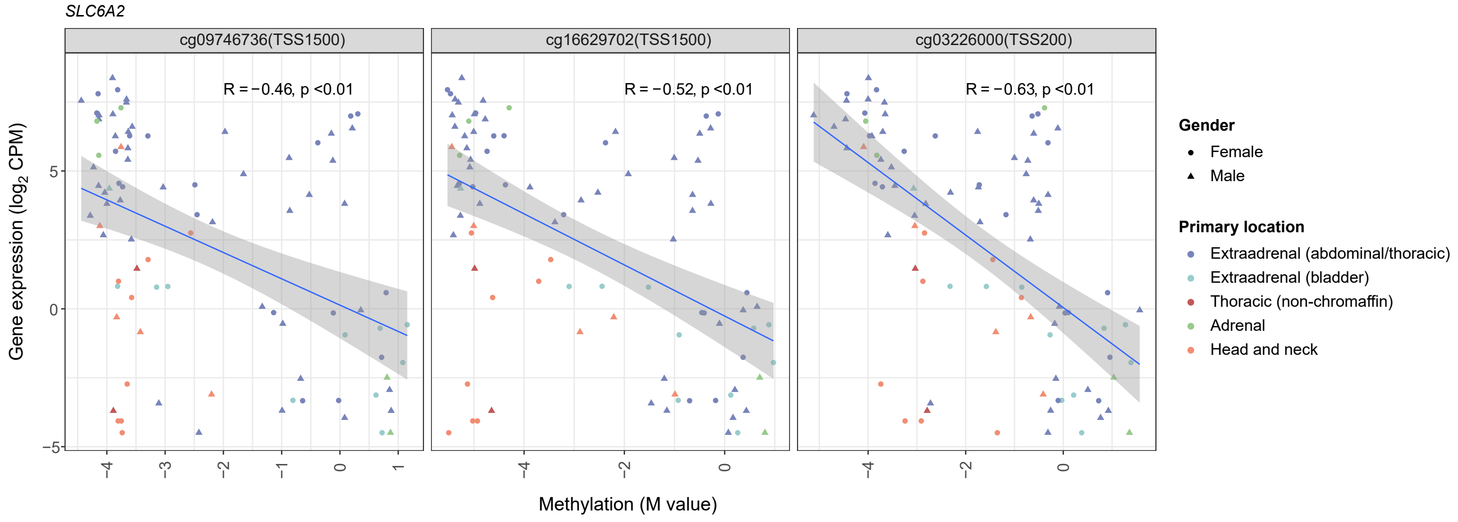

Supplementary Figure 9 Methylation proximal to the gene transcription start site correlates with gene expression for *SLC6A2*
Methylation (x-axis, M-values) of probes located 200 bp (TSS200) or 1500 bp (TSS1500) upstream of the transcription start site versus gene expression (y-axis, log2 CPM) of *SLC6A2*. A linear regression line (blue line), 95% confidence interval (shaded grey area), and correlation coefficient (R) for a Spearman correlation are provided (n = 88).

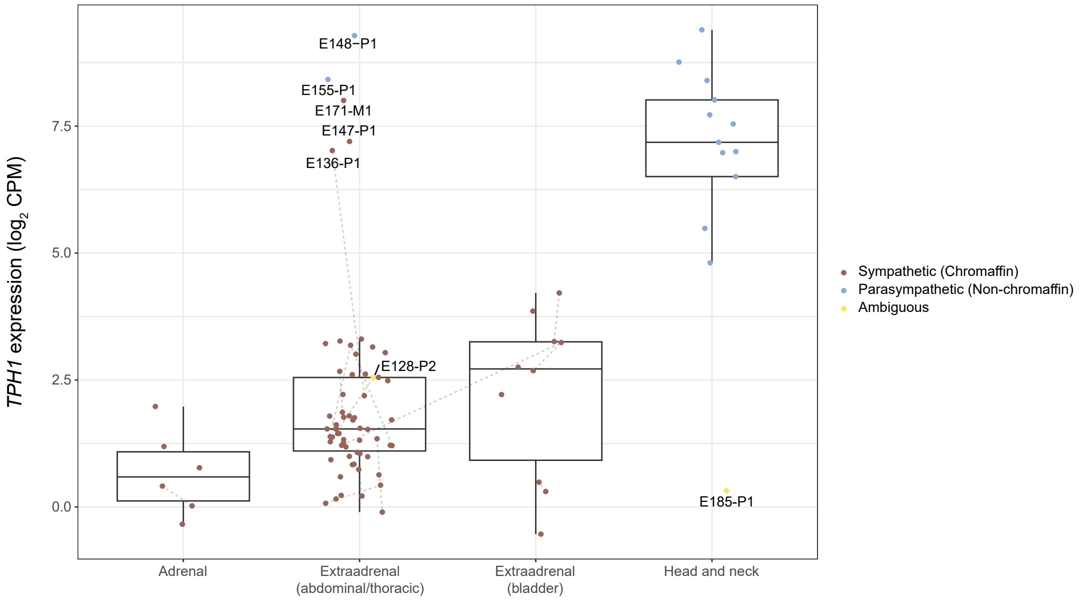

Supplementary Figure 10 Expression of *TPH1* in HN-PG relative to sympathetic PCPG
TPH1 expression (y-axis, log2 CPM) versus site of primary disease (x-axis). Each point represents a tumour (n=91), point colour indicates whether the tumour clustered with parasympathetic (non-chromaffin) or sympathetic (chromaffin) type tumours by UMAP clustering of WTS. Outliers and samples that clustered ambiguously are labelled. Paired samples from an individual patient are joined by a dotted line. The hinges of each boxplot correspond to the first and third quartiles and the median value is marked. The whiskers extend to the largest and smallest value no greater than 1.5 times the interquartile range above or below the upper and lower hinges, respectively.

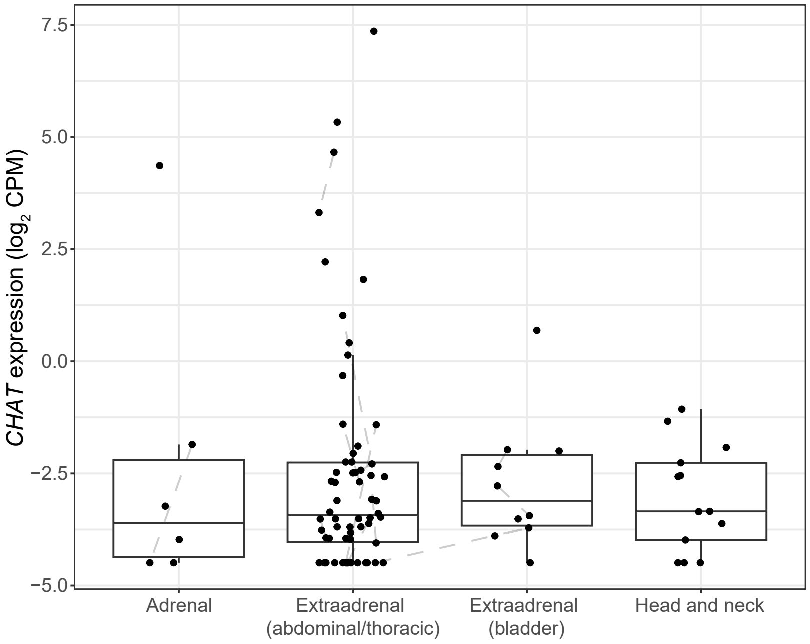

Supplementary Figure 11 Expression of Choline O-Acetyltransferase (*CHAT*)
CHAT expression (y-axis, log2 CPM) across the cohort. Samples (n=91) are stratified by the anatomical location of the tumour or, in the case of a metastasis, the associated primary tumour. Samples from the same patient are joined by a dotted line. The hinges of each boxplot correspond to the first and third quartiles and the median value is marked. The whiskers extend to the largest and smallest value no greater than 1.5 times the interquartile range above or below the upper and lower hinges, respectively.

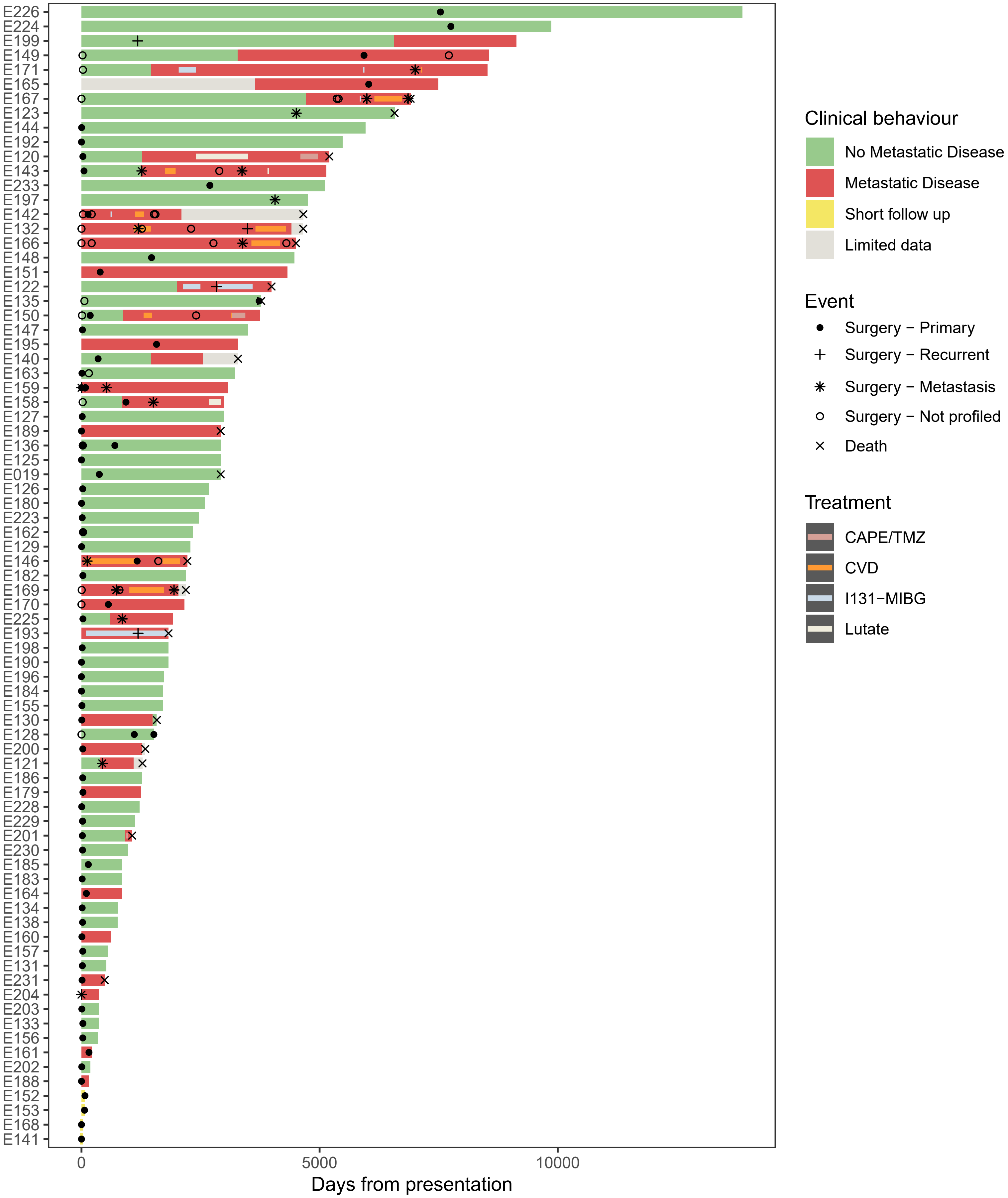

Supplementary Figure 12 Clinical timeline of treatment
Clinical timelines for each patient (y-axis) covering the days (x-axis) from initial presentation until death or cessation of follow-up. Surgical events and patient death are indicated by points along the timeline. Disease progression or lack of clinical data is indicated by the colour of the timeline (thick bar). Treatment modalities are indicated by the colour of the internal bar.

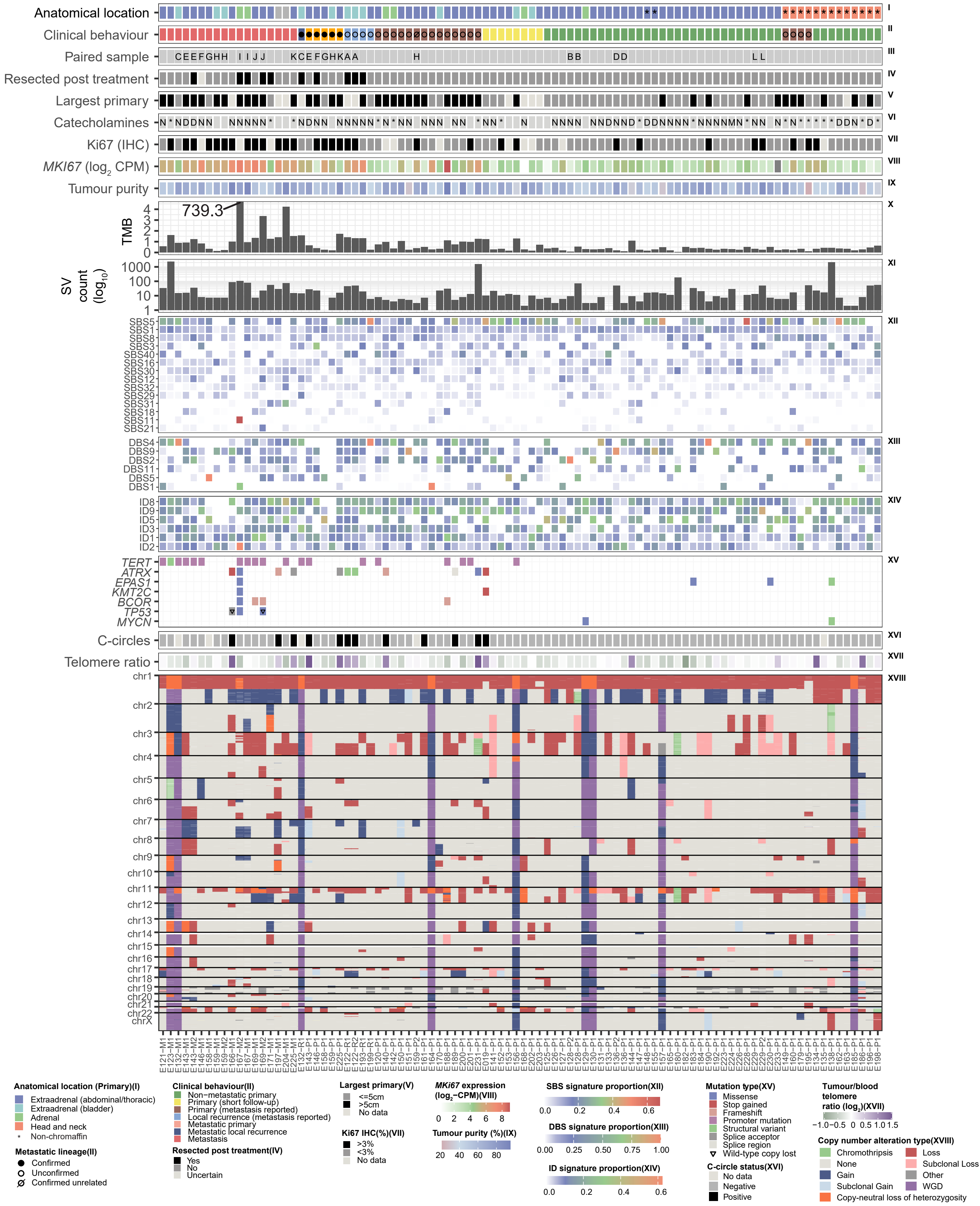

Supplementary Figure 13 Summary of clinical and genomic features of the cohort
(I) Anatomical location of the tumour or, in the case of a metastasis, the associated primary tumour. **(II)** Clinical behaviour of the specimen indicating whether the tumour specimen was derived from a non-metastatic primary, a primary or local recurrence from a patient that had metastatic disease but could not be confirmed as the source of metastasis (primary/local-recurrence - metastasis reported), a primary or local recurrence that was confirmed to be the source of metastatic disease (metastatic primary/local-recurrence), a metastasis, or if the primary had insufficient follow up for classification (primary - short follow up). **(III)** An identifier linking samples from the same patient. **(IV)** Indicates if the sample was resected after a cytotoxic treatment regime **(V)** Status of the largest primary in the patient as larger (black) or smaller (grey) than 5cm. **(VI)** The biochemical profile of the tumour: N=Norepinephrine, E=Epinephrine, D=Dopamine, M=Mixed, *=Biochemically silent. **(VII)** Status of the tumour as having a percentage of Ki67 positive cells greater (black) of less (grey) than 3%. **(VIII)** Expression of MKI67 (log2 CPM) **(IX)** The tumour purity estimate provided by PURPLE. **(X)** Tumour mutation burden in mutations per megabase (y-axis), the axis has been truncated to exclude one outlier (red text). **(XI)** Structural variant count (y-axis, log10). **(XII, XIII, XIV)** Single and doublet base substitution, and insertion/deletion signatures. Colour indicates the proportion of variants attributed to a given signature as a proportion of all variants in a given sample. SBS, DBS, and ID signatures shown are those that had a proportion contribution greater than 15% and and absolute contribution greater than 500, 10, and 50, respectively, in at least one sample. **(XV)** Recurrently mutated genes across the cohort. Colour indicates the consequence of the mutation, loss of the wild-type copy is indicated by a downward facing triangle. **(XVI)** Status of the tumour as either C-circle positive (black) or negative (dark grey) based on the C-circle assay. **(XVII)** Heatmap showing the ratio (log2) of telomeric content in the tumour to the germline control. **(XVII)** Copy number status along each chromosome, bar colour indicates the copy number classification for a given segment.

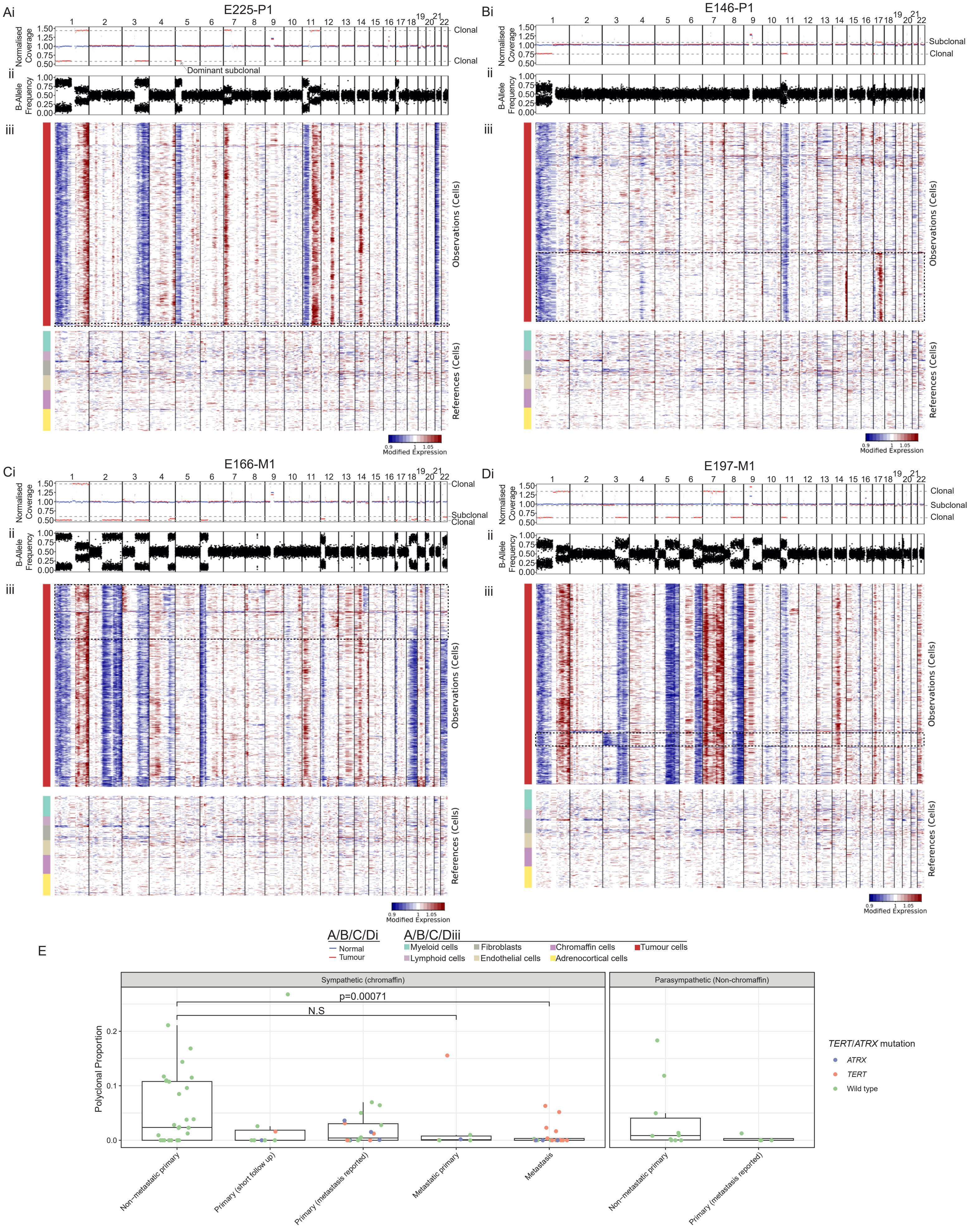

Supplementary Figure 14 Subclonal populations in PCPG

(A/B/C/D) (i) Normal and tumour normalised sequencing coverage and **(ii)** B-allele frequencies obtained from bulk WGS. **(iii)** Copy number status imputed by inferCNV from single-nuclei RNA sequencing. **(E)** Polyclonality scores assigned by PURPLE indicating the proportion of copy number regions that are more than 0.25 from a whole copy number. Statistical significance was assessed with a Student's t-test for the groups indicated (n = { Chromaffin - Non-metastatic primary: 28; Chromaffin - Metastatic primary: 5; Chromaffin - Metastasis: 18, All = 92 }). The hinges of each boxplot correspond to the first and third quartiles and the median value is marked. The whiskers extend to the largest and smallest value no greater than 1.5 times the interquartile range above or below the upper and lower hinges, respectively.

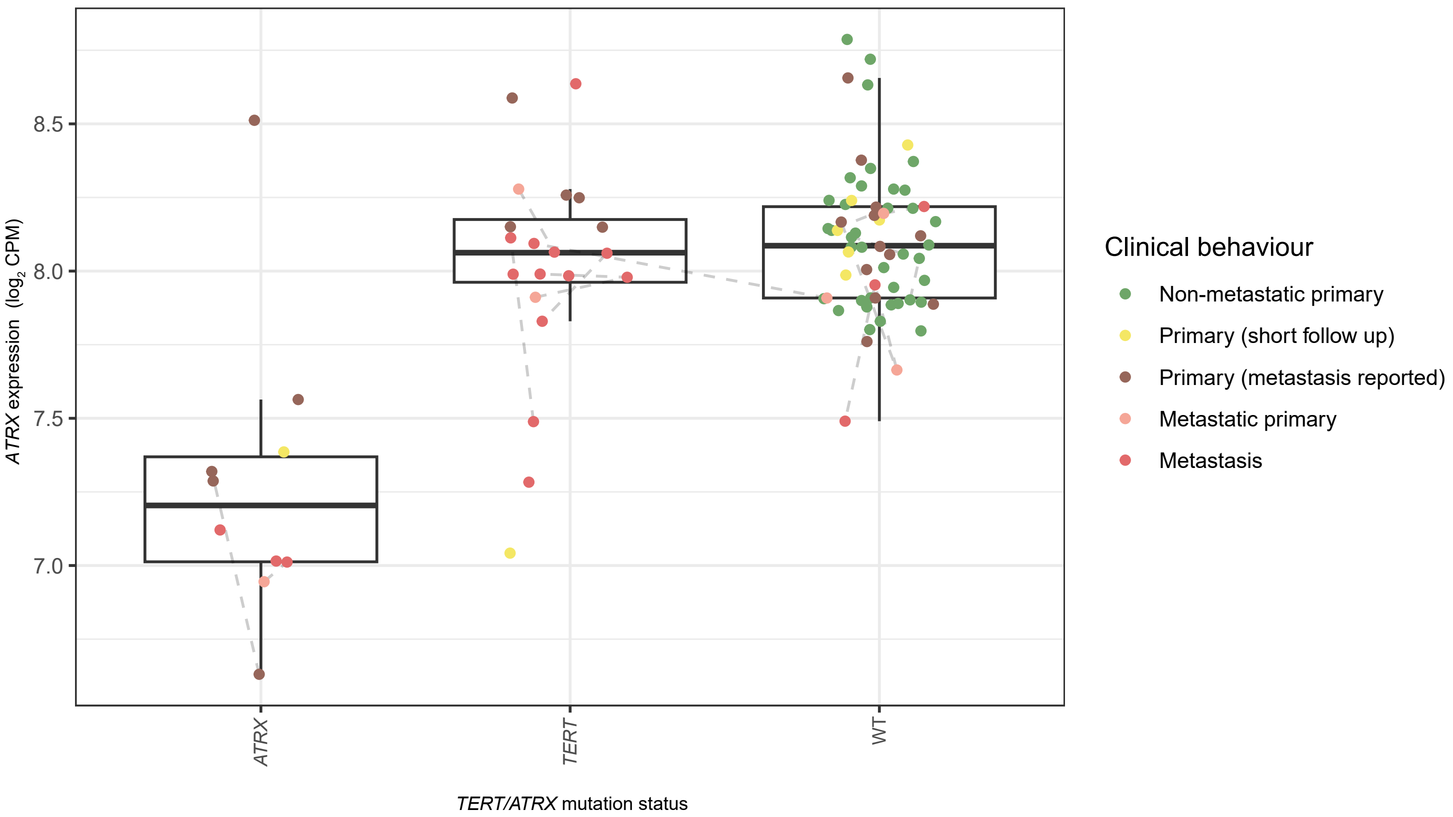

Supplementary Figure 15 *ATRX* expression is reduced in *ATRX* mutant tumours
ATRX expression (y-axis) with samples (n=91) stratified by the presence of an *ATRX* mutation, a *TERT* mutation, or no mutation (wild-type, WT). Each point represents a tumour, point colour indicates the clinical behaviour of the tumour. Tumours from the same patient are joined by a dotted line. The hinges of each boxplot correspond to the first and third quartiles and the median value is marked. The whiskers extend to the largest and smallest value no greater than 1.5 times the interquartile range above or below the upper and lower hinges, respectively.

A

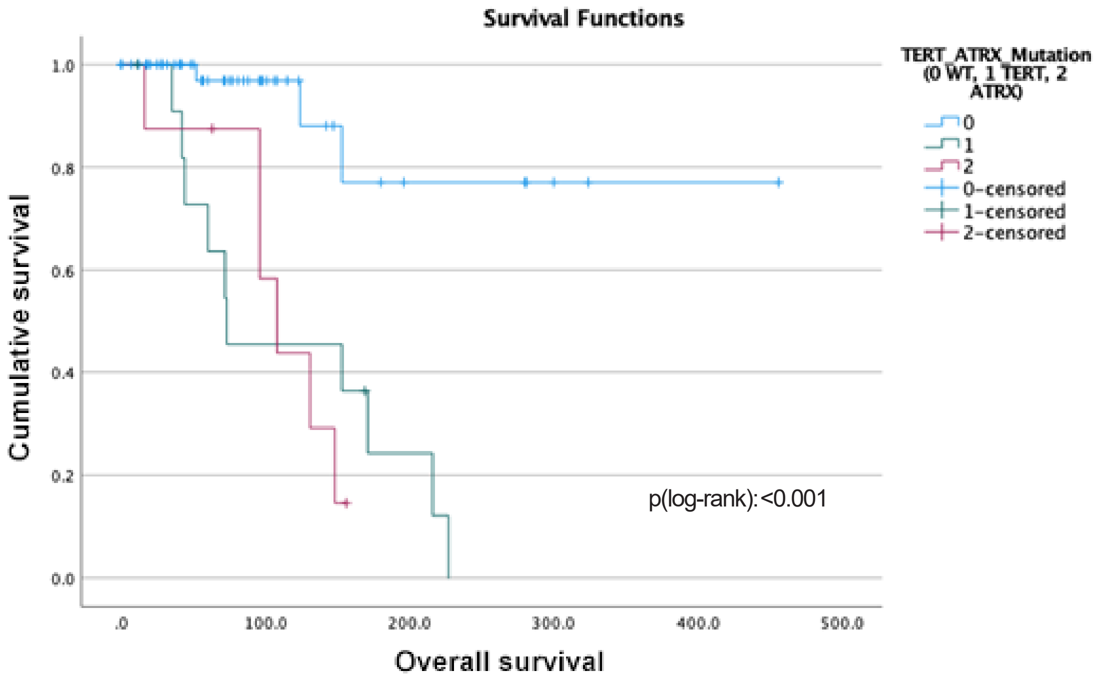

B

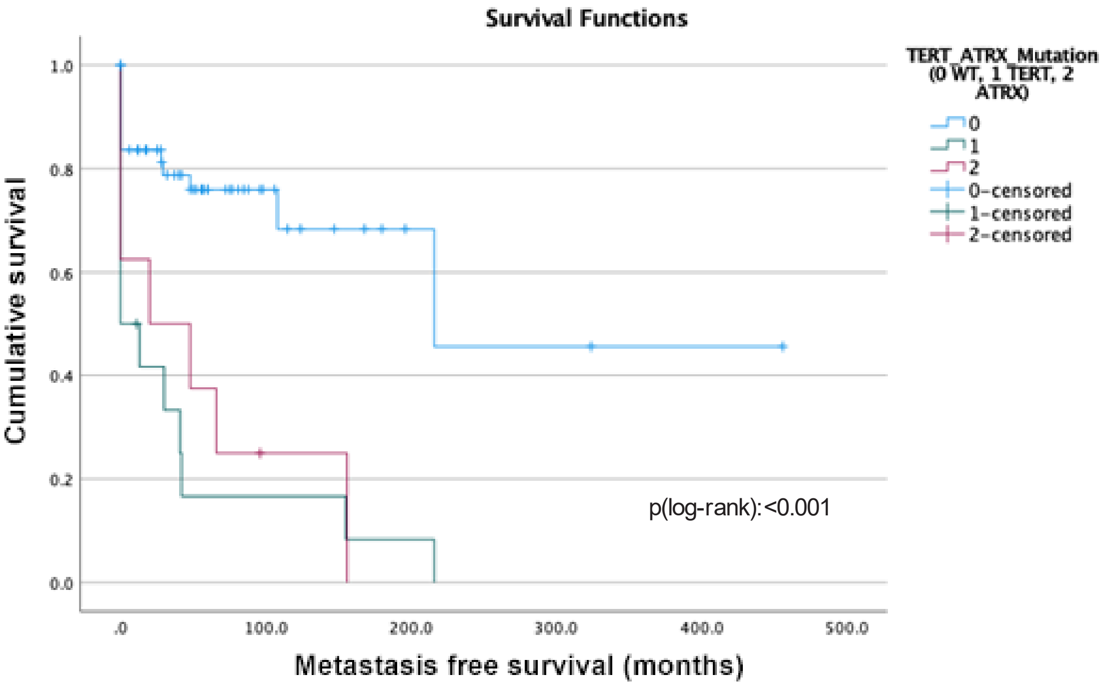

Supplementary Figure 16 Kaplan-Meier analyses for *TERT*/*ATRX* mutational status
Kaplan-Meier analyses for (a) overall survival (months) and (b) metastasis-free survival according to *TERT* (n=20), *ATRX* (n=10) or wild type (WT, n=63) mutational status. Hypothesis testing was performed using a log-rank (Mantel-Cox) test.

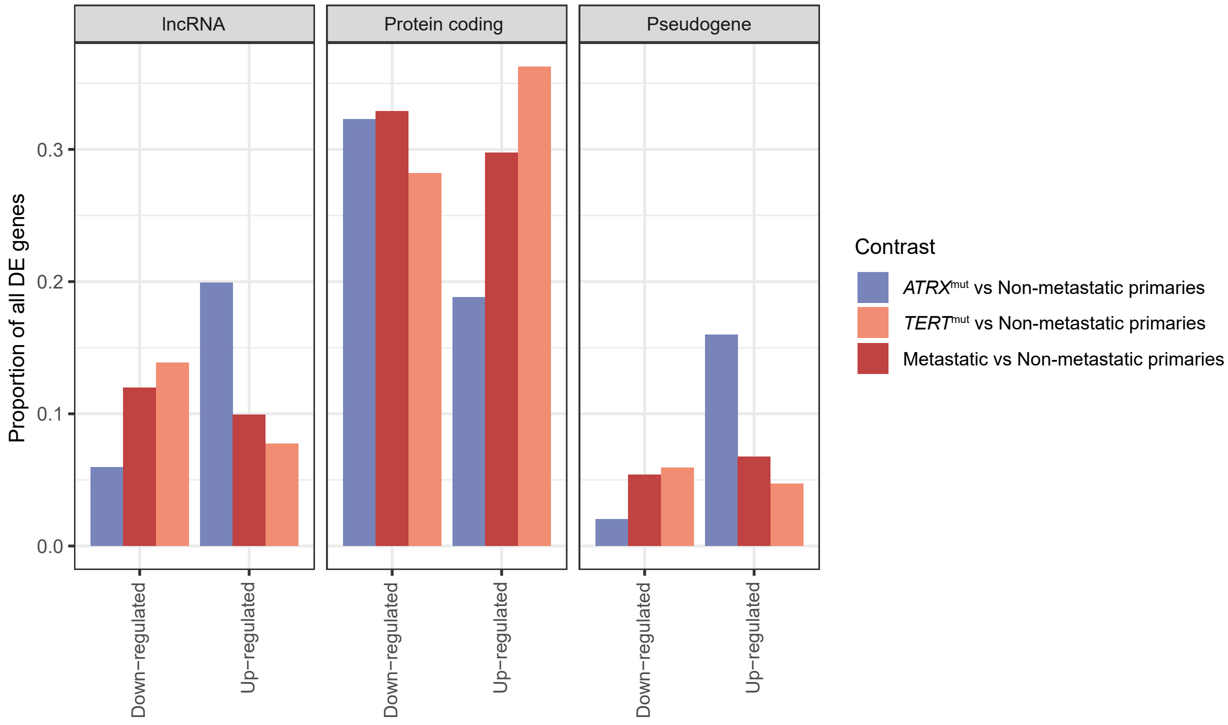

Supplementary Figure 17 Gene biotype distribution in differentially expressed genes in *ATRX* and *TERT*-altered tumours
Differential expression analysis was performed between non-metastatic primary tumours (n=21) and *ATRX*-altered (n=9), *TERT*-altered (n=15), or all metastatic tumours (n=35). Significant genes (adjusted p-value < 0.05) were categorised as up-regulated (log fold change > 0) or down-regulated (log fold change < 0) and annotated with their biotype. The proportion of genes (y-axis) was computed as the total number genes in each category (up/down) for each biotype divided by the total number significant genes for a given contrast.

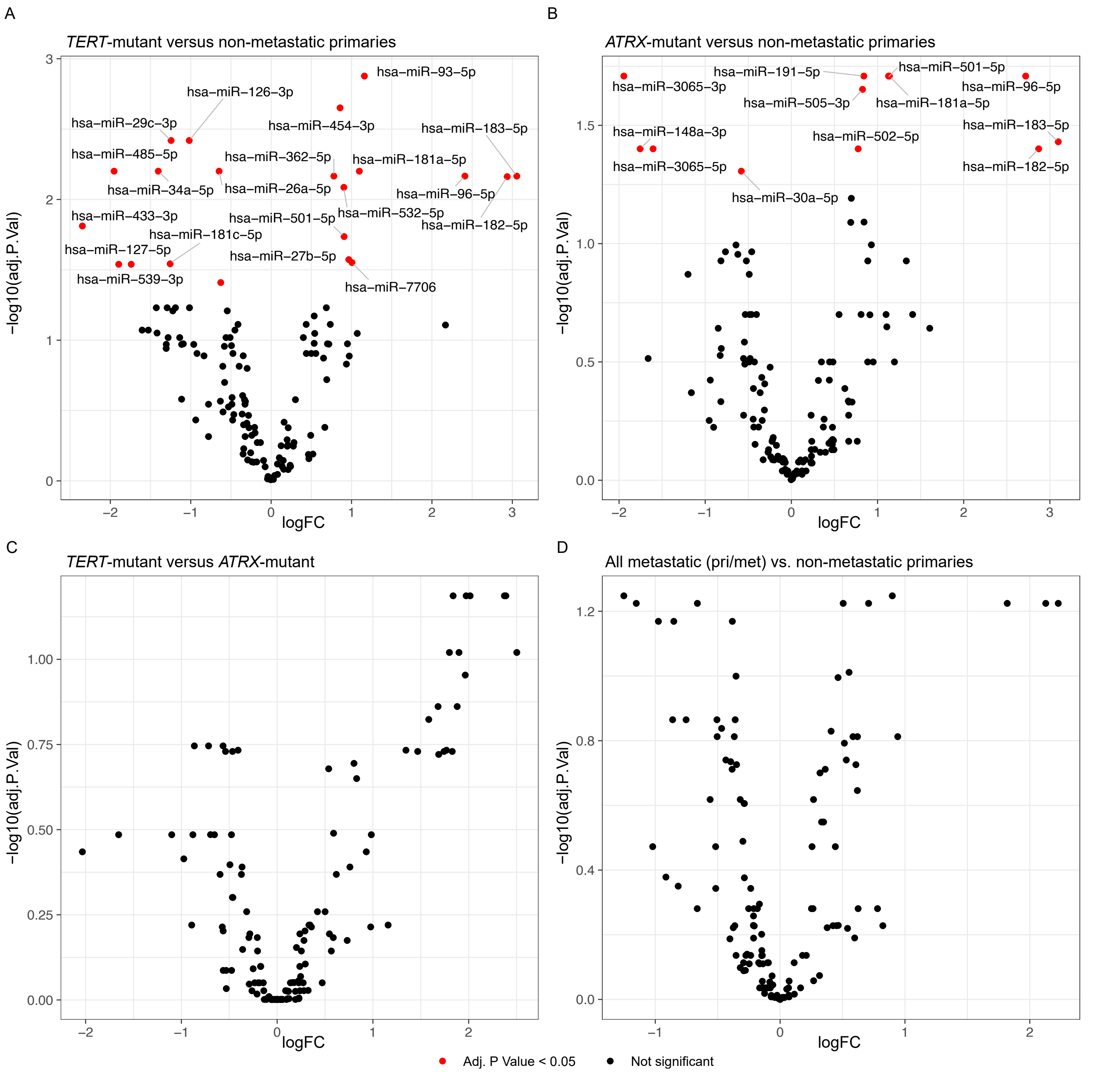

Supplementary Figure 18 Differential expression analysis of small-RNA-seq in SDHB-mutant sympathetic PCPG
Differential expression analysis of small-RNA-seq was performed using the limma/voom pipeline for the following contrasts: **(A)** *TERT*-mutant primary and metastatic tumours (n=12) versus wild-type non-metastatic primary tumours (n=21), **(B)** *ATRX*-mutant primary and metastatic tumours (n=9) versus wild-type non-metastatic primary tumours (n=21), **(C)** *TERT*-mutant primary and metastatic tumours (n=12) versus *ATRX*-mutant primary and metastatic tumours (n=9), and **(D)** all metastases and metastatic primary tumours (n=31) versus non-metastatic primary tumours (n=21). Genes reaching statistical significance (FDR adjusted p-value < 0.05) are indicated in red.

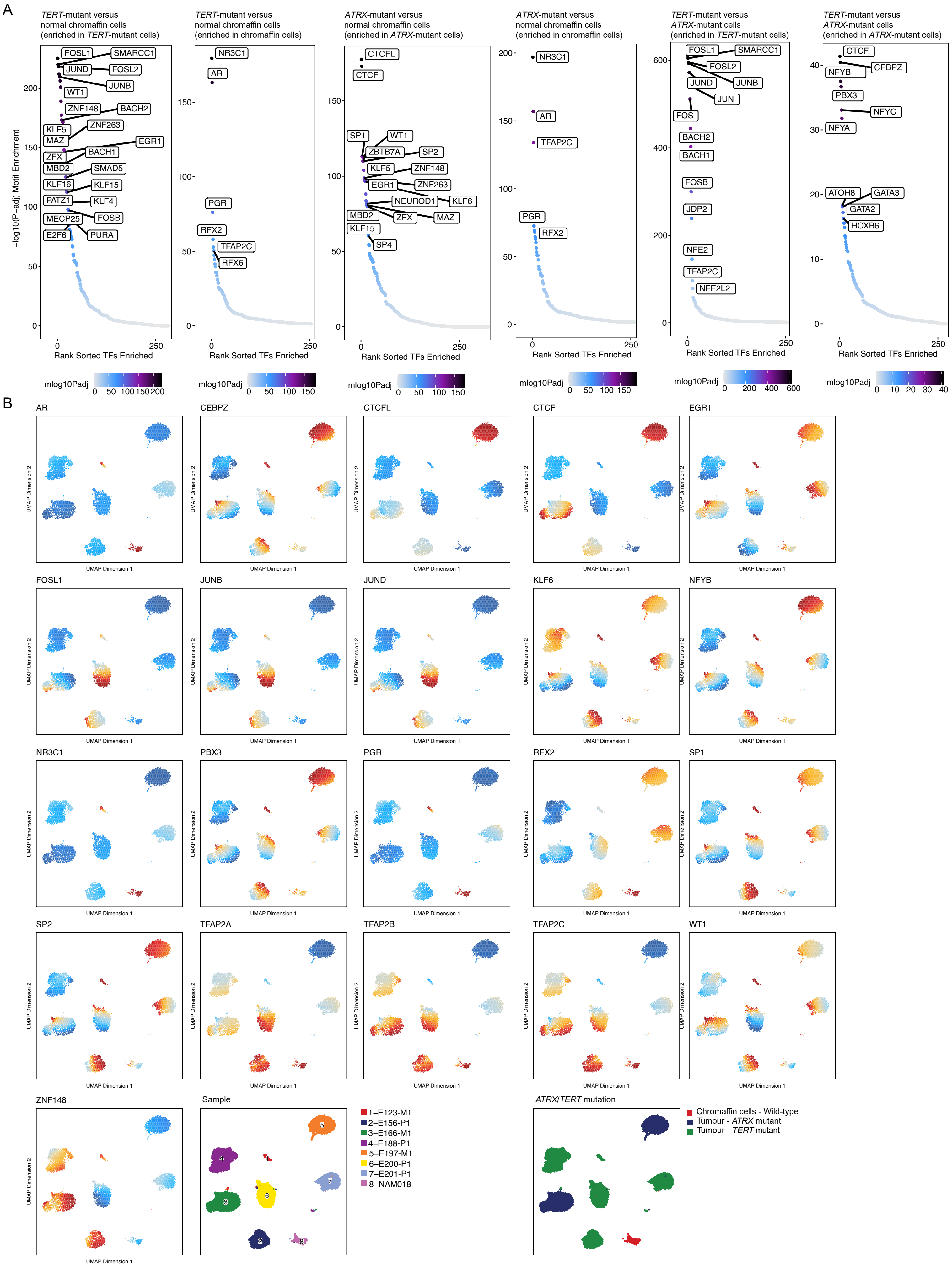

Supplementary figure 19 Transcription factor binding motif enrichment analysis

Single-nuclei ATAC seq peaks were analysed for transcription factor binding motifs. (A) Enrichment analysis was performed contrasting *TERT*-mutant, *ATRX*-mutant, and normal chromaffin cells. (B) UMAP clustering of cells from single nuclei ATAC-seq. Colour indicates relative depletion (blue) or enrichment (red) of the indicated binding motif. nSamples=8, nCells= {E123-M1: 165, E156-P1: 1600, E166-M1: 2688, E188-P1: 3129, E197-M1: 4000, E200-P1: 2377, E201-P1: 1983, NAM018: 285, Normal chromaffin cells - wild-type: 289, Tumour - *ATRX* mutant: 6687, Tumour - *TERT* mutant: 9251}.

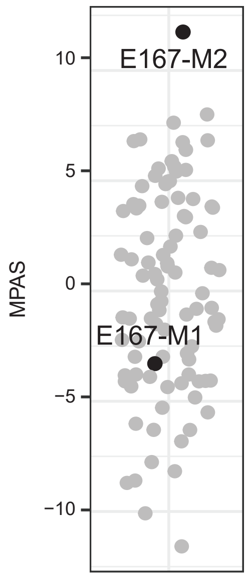

Supplementary Figure 20 MAP Kinase pathway activity score (MPAS)
The MPAS was computed based on the expression of nine MAP Kinase pathway genes (*SPRY2, SPRY4, ETV4, ETV5, DUSP4, DUSP6, CCND1, EPHA2, EPHA4*). First, the expression of these genes across the cohort were centred around their mean and scaled (Z-score). Then, for each sample, the MPAS score (y-axis) was computed as the sum of the Z-scores across the nine genes.

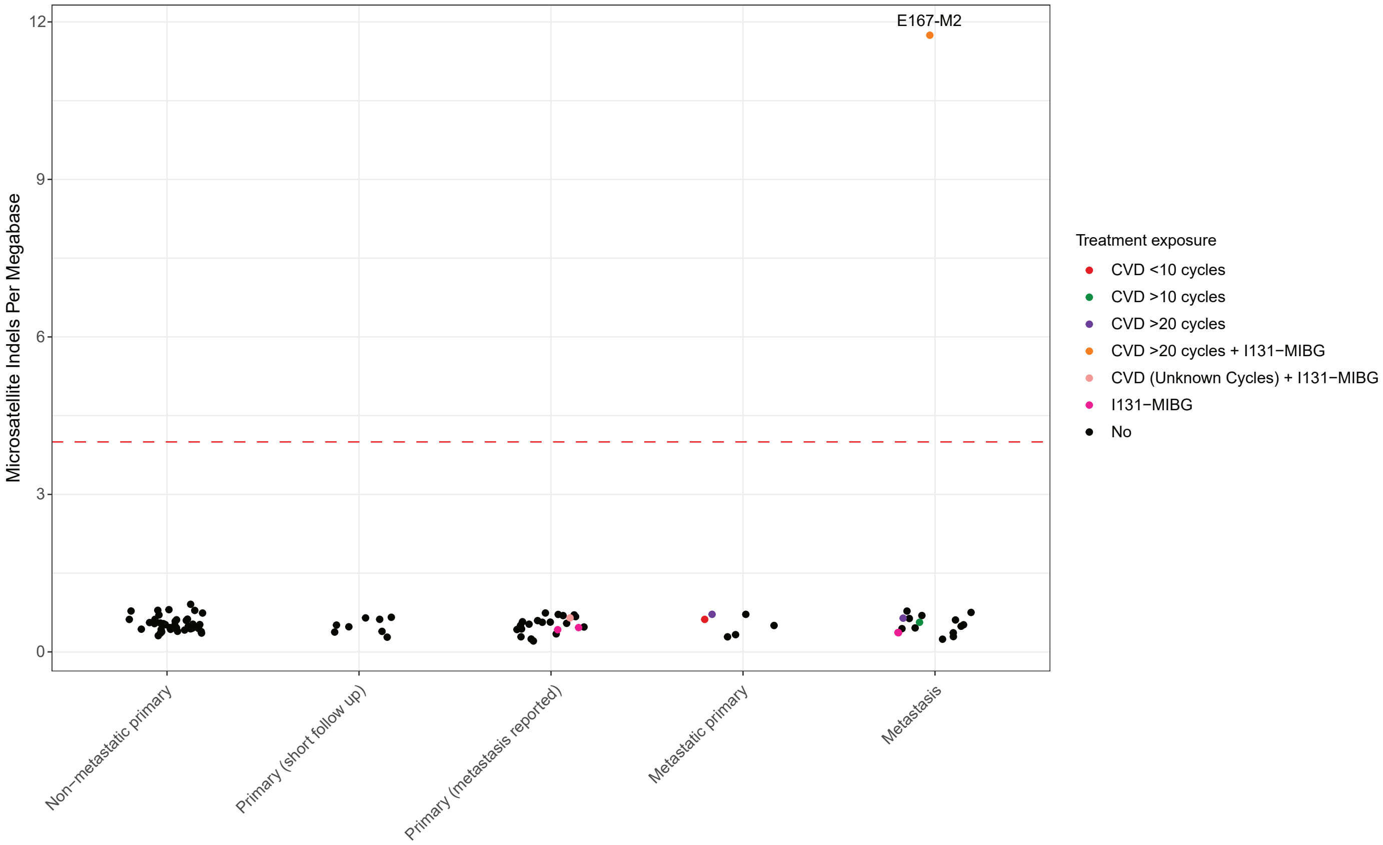

Supplementary Figure 21 Insertion/Deletions at microsatellite sites
The number of insertion/deletion events at microsatellite sites (y-axis) was computed by PURPLE. Each point represents a tumour, points are coloured by exposure to cytotoxic chemotherapy and tumours are stratified by clinical behaviour (x-axis). A dotted red line indicates the cutoff threshold for microsatellite instability.

Supplementary Table. 1

Cox proportional hazards regression was performed to assess impact of *TERT* or *ATRX* mutations, primary tumour size ≥5 cm, age at diagnosis or sex on overall or metastasis-free survival. For overall survival, 63 cases were included in the analysis (17 disease-specific deaths) and for metastasis-free survival, 71 cases were included in analysis (31 with metastatic progression). Significant hazard ratios (HR) were those with a p-value less than 0.05 using the Wald test and are marked with bold text.

	**Overall survival**			**Metastasis-free survival**		
**Variable**	**HR**	**95%CI**	**Significance**	**HR**	**95%CI**	**Significance**
TERT or *ATRX*	**4.308**	1.031-18.011	0.045	**2.953**	1.183-7.371	0.020
Primary tumour ≥5 cm	**5.294**	1.172-29.913	0.030	**3.086**	1.177-8.090	0.022
Age at diagnosis (y)	1.034	0.997-1.072	0.068	0.976	0.953-1.001	0.058
Female sex	0.312	0.096-1.013	0.053	1.078	0.520-2.234	0.840

Supplementary table 2: Bcbio-nextgen pipeline component versions

Program name	Version
bamtofastq	2.0.87
bamtools	2.4.0
bcbio-nextgen	1.2.4-76d5c4ba
bcbio-variation	0.2.6
bcftools	1.9
bedtools	2.27.1
biobambam	2.0.87
bioconductor-bubbletree	2.6.0
bowtie2	2.4.1
break-point-inspector	1.5
bwa	0.7.17
cnvkit	0.9.7
cufflinks	2.2.1
cutadapt	2.1
ensembl-vep	100.4
fastqc	0.11.8
featureCounts	v2.0.1
fgbio	1.3.0
freebayes	1.1.0.46
gatk	3.8
gatk4	4.1.8.1
gemini	0.30.2
grabix	0.1.8
hisat2	2.2.0
htseq	0.9.1
lumpy-sv	0.3.1
manta	1.6.0
metasv	0.4.0
mirdeep2	2.0.0.7
multiqc	1.9
novoalign	4.02.02
novosort	V2.02.00
oncofuse	1.1.1
phylowgs	20181105
picard	2.23.4
platypus-variant	0.8.1.2
qualimap	2.2.2d
rapmap	0.6.0
rtg-tools	3.11
sailfish	0.10.1
salmon	1.3.0
sambamba	0.7.1
samblaster	0.1.26
samtools	1.9
scalpel	0.5.4
seq2c	1.3
seqbuster	3.5
snpeff	4.3.1t
star	2.6.1d
umis	1.0.7
vardict	2019.06.04
vardict-java	1.8.2
varscan	2.4.4
vcflib	1.0.0_rc2
vt	2015.11.10
wham	1.8.0.1.2017.05.03
