# Supplementary material for: Multi-omic analysis of SDHB-deficient pheochromocytomas and paragangliomas identifies metastasis and treatment-related molecular profiles
Source: Nat Commun. 2025 Mar 17;16:2632. doi: 10.1038/s41467-025-57595-y (PMC11914184; doi:10.1038/s41467-025-57595-y)
Supplement: Supplementary file 2 — Description of Additional Supplementary Information [file 41467_2025_57595_MOESM2_ESM.docx]

**Description of Additional Supplementary Files**

File Name: Supplementary Data 1

Description: Patient characteristics

File Name: Supplementary Data 2

Description: Tumour specimen annotation

File Name: Supplementary Data 3

Description: Differential expression between sympathetic (chromaffin) and parasympathetic (non-chromaffin) PCPG from WTS

File Name: Supplementary Data 4

Description: Differential expression between sympathetic (chromaffin) and parasympathetic (non-chromaffin) PCPG from small RNA sequencing

File Name: Supplementary Data 5

Description: Somatic coding mutations in SDHB-related PCPG

File Name: Supplementary Data 6

Description: Differential expression between TERT-altered, ATRX-altered, and nonmetastatic primary PCPG from WTS

File Name: Supplementary Data 7

Description: Differential expression between TERT-altered, ATRX-altered, and nonmetastatic primary PCPG from small RNA sequencing
