# Supplementary material for: Multi-omic analysis of SDHB-deficient pheochromocytomas and paragangliomas identifies metastasis and treatment-related molecular profiles
Source: Nat Commun. 2025 Mar 17;16:2632. doi: 10.1038/s41467-025-57595-y (PMC11914184; doi:10.1038/s41467-025-57595-y)
Supplement: Supplementary file 10 — Reporting Summary [file 41467_2025_57595_MOESM10_ESM.pdf]

nature portfolio

Corresponding author(s): Richard Tothill

Last updated by author(s): Feb 3, 2025

Reporting Summary

Nature Portfolio wishes to improve the reproducibility of the work that we publish. This form provides structure for consistency and transparency in reporting. For further information on Nature Portfolio policies, see our Editorial Policies and the Editorial Policy Checklist.

Statistics

For all statistical analyses, confirm that the following items are present in the figure legend, table legend, main text, or Methods section.

n/a	Confirmed	
☐	☒	The exact sample size (*n*) for each experimental group/condition, given as a discrete number and unit of measurement
☒	☐	A statement on whether measurements were taken from distinct samples or whether the same sample was measured repeatedly
☐	☒	The statistical test(s) used AND whether they are one- or two-sided<br>*Only common tests should be described solely by name; describe more complex techniques in the Methods section.*
☐	☒	A description of all covariates tested
☐	☒	A description of any assumptions or corrections, such as tests of normality and adjustment for multiple comparisons
☐	☒	A full description of the statistical parameters including central tendency (e.g. means) or other basic estimates (e.g. regression coefficient) AND variation (e.g. standard deviation) or associated estimates of uncertainty (e.g. confidence intervals)
☐	☒	For null hypothesis testing, the test statistic (e.g. *F*, *t*, *r*) with confidence intervals, effect sizes, degrees of freedom and *P* value noted<br>*Give P values as exact values whenever suitable.*
☒	☐	For Bayesian analysis, information on the choice of priors and Markov chain Monte Carlo settings
☒	☐	For hierarchical and complex designs, identification of the appropriate level for tests and full reporting of outcomes
☒	☐	Estimates of effect sizes (e.g. Cohen's *d*, Pearson's *r*), indicating how they were calculated

Our web collection on statistics for biologists contains articles on many of the points above.

Software and code

Policy information about availability of computer code

Data collection	No data collection software was used
Data analysis	____Third party tools____<br>bamtofastq 2.0.87<br>bamtools 2.4.0<br>bcbio-nextgen 1.2.4-76d5c4ba<br>bcbio-variation 0.2.6<br>bcftools 1.9<br>bedtools 2.27.1<br>biobambam 2.0.87<br>bioconductor-bubbletree 2.6.0<br>bowtie2 2.4.1<br>break-point-inspector 1.5<br>bwa 0.7.17<br>cnvkit 0.9.7<br>cufflinks 2.2.1<br>cutadapt 2.1<br>ensembl-vep 100.4<br>fastqc 0.11.8

featureCounts v2.0.1
fgbio 1.3.0
freebayes 1.1.0.46
gatk 3.8
gatk4 4.1.8.1
gemini 0.30.2
grabix 0.1.8
hisat2 2.2.0
htseq 0.9.1
lumpy-sv 0.3.1
manta 1.6.0
metasv 0.4.0
mirdeep2 2.0.0.7
multiqc 1.9
novoalign 4.02.02
novosort V2.02.00
oncofuse 1.1.1
phylowgs 20181105
picard 2.23.4
platypus-variant 0.8.1.2
qualimap 2.2.2d
rapmap 0.6.0
rtg-tools 3.11
sailfish 0.10.1
salmon 1.3.0
sambamba 0.7.1
samblaster 0.1.26
samtools 1.9
scalpel 0.5.4
seq2c 1.3
seqbuster 3.5
snpeff 4.3.1t
star 2.6.1d
umis 1.0.7
vardict 2019.06.04
vardict-java 1.8.2
varscan 2.4.4
vcflib 1.0.0_rc2
vt 2015.11.10
wham 1.8.0.1.2017.05.03
GRIDSS (version 2.8.0)
GRIPPS (version 1.11)
PURPLE (version 3.1)
COBALT (version 1.11)
AMBER (version 3.5)
TelomereHunter (version 1.1.0)
HTSeq-count (version 0.11.3)
edgeR (version 3.42.4)
limma (version 3.56.2)
featurecounts (version 1.6.3)
minfi (version 1.46.0)
MissMethyl (version 1.34.0)
DMRcate (version 2.14.1)
TCGAbiolinks (version 2.29.1)
CellRanger (version 7.2.0)
SCRUBLET (version 0.2.3)
Seurat (version 4.3.0.1)
cellranger-atac (V2.0.0).
Signac (version 1.12.9004)
MACS2 (version 2.2.9.1)
Illumina DRAGEN TruSight Oncology 500 ctDNA Analysis Software v1.2
R (version 4.3.1)
ArchR (version 1.02)
Pairtree (version 1.0.1)
___Custom code ____
https://github.com/UMCCR-RADIO-Lab/a5_sdhb_pcpg

For manuscripts utilizing custom algorithms or software that are central to the research but not yet described in published literature, software must be made available to editors and reviewers. We strongly encourage code deposition in a community repository (e.g. GitHub). See the Nature Portfolio guidelines for submitting code & software for further information.

Data

Policy information about availability of data

All manuscripts must include a data availability statement. This statement should provide the following information, where applicable:

- Accession codes, unique identifiers, or web links for publicly available datasets
- A description of any restrictions on data availability
- For clinical datasets or third party data, please ensure that the statement adheres to our policy

Raw FASTQ files for WGS, WTS, small-RNA-sequencing, sn-ATAC-sequencing, sn-RNA-sequencing, as well as GRCh38 aligned CRAM files for WGS (accession EGAS50000000346 [https://ega-archive.org/studies/EGAS50000000346]), and IDAT files for Illumina EPIC methylation array profiling (accession EGAS00001007844 [https://ega-archive.org/studies/EGAS00001007844]) are available from the European-genome-phenome (EGA) archive. As genomic data represent potentially identifying information, these data are under restricted access controlled by the Data Access Committee at the University of Melbourne Centre for Cancer Research. Access to sequence read-level data or germline variant data will require evidence of institutional human research ethics committee approval. Access can be obtained by application through the EGA portal and applications will be processed within four to six weeks. If approved, data access will be granted in perpetuity. Unrestricted access to a data package containing non-identifiable data from tertiary analysis including somatic small and structural variant calls, copy-number analysis, RNA-fusion calls, and gene expression matrices from bulk and single nuclei analysis is available from Figshare (https://doi.org/10.6084/m9.figshare.25792479). RNA-seq, Illumina Infinium HumanMethylation450 arrays, and small-RNA-seq data previously made available by Fishbein et al. (1) was obtained from the National Computational Infrastructure's Genomic Data Commons. Bulk RNA-seq data previously published by Flynn et al. (2) and snRNA data published by Zethoven et al. was obtained from EGA (EGAS00001005861 [https://ega-archive.org/studies/EGAS00001005861]) (3). Small-RNA-seq previously published by Castro-Vega et al were obtained from ArrayExpress (E-MTAB-2833 [https://www.ebi.ac.uk/arrayexpress/experiments/E-MTAB-2833]) (4). Illumina Infinium HumanMethylation450 and HumanMethylation27 arrays previously made available by Letouzé et al. were obtained from the Gene Expression Omnibus (accession GSE39198 [https://www.ncbi.nlm.nih.gov/geo/query/acc.cgi?acc=GSE39198], and GSE43293 [https://www.ncbi.nlm.nih.gov/geo/query/acc.cgi?acc=GSE43293])(5). The remaining data are available within the Article, Supplementary Information, or Source Data file.

References:

(1) Fishbein, L., et al. Comprehensive Molecular Characterization of Pheochromocytoma and Paraganglioma. Cancer Cell 31, 181-193 (2017).

(2) Flynn, A., et al. The genomic landscape of phaeochromocytoma. J Pathol 236, 78-89 (2015).

(3) Zethoven, M., et al. Single-nuclei and bulk-tissue gene-expression analysis of pheochromocytoma and paraganglioma links disease subtypes with tumor microenvironment. Nat Commun 13, 6262 (2022).

(4) Castro-Vega, L.J., et al. Multi-omics analysis defines core genomic alterations in pheochromocytomas and paragangliomas. Nat Commun 6, 6044 (2015).

(5) Letouze, E., et al. SDH mutations establish a hypermethylator phenotype in paraganglioma. Cancer Cell 23, 739-752 (2013).

Research involving human participants, their data, or biological material

Policy information about studies with human participants or human data. See also policy information about sex, gender (identity/presentation), and sexual orientation and race, ethnicity and racism.

Reporting on sex and gender	Patient sex is not included in the clinical characteristics table but was used as a factor in a Cox proportional hazards regression and differential methylation analysis. The findings of this study apply equally to both sexes
Reporting on race, ethnicity, or other socially relevant groupings	Race or ethnicity is not considered in the study
Population characteristics	Patient age at diagnosis is included in the clinical characteristics table and as a factor in a Cox proportional hazards regression
Recruitment	Patients were recruited at 11 sites under protocols approved by their respective institutional review boards with written informed consent without any compensation. Patient recruitment sites included those forming the American-Australian-Asian-Adrenal-Alliance (A5) International Research Consortium including the Peter MacCallum Cancer Centre (Australia), the Kolling Institute Neuroendocrine Tumour Bank under a protocol approved at North Sydney Local Health District (Australia), National Institute of Health (USA), University of Colorado (USA), University of Texas Health Science Center at San Antonio (USA), University of Florida (USA), University of Michigan (USA), Centre hospitalier de l'université de Montréal (Canada), as well as four non-A5 sites at Tufts Medical Centre (USA), Waikato Hospital (New Zealand), the National Cancer Centre (Singapore), and Uppsala University (Sweden)
Ethics oversight	Study protocol approval was obtained from the ethics boards at the relevant points of sample collection including Peter MacCallum Cancer Centre (Australia), the Kolling Institute Neuroendocrine Tumour Bank via North Sydney Local Health District (Australia), National Institute of Health (USA), University of Colorado (USA), University of Texas Health Science Center at San Antonio (USA), University of Florida (USA), University of Michigan (USA), Centre hospitalier de l'université de Montréal (Canada), Tufts Medical Centre (USA), Waikato Hospital (New Zealand), the National Cancer Centre (Singapore), and Uppsala University (Sweden)

Note that full information on the approval of the study protocol must also be provided in the manuscript.

Field-specific reporting

Please select the one below that is the best fit for your research. If you are not sure, read the appropriate sections before making your selection.

☒ Life sciences ☐ Behavioural & social sciences ☐ Ecological, evolutionary & environmental sciences

For a reference copy of the document with all sections, see nature.com/documents/nr-reporting-summary-flat.pdf

Life sciences study design

All studies must disclose on these points even when the disclosure is negative.

Sample size	Sample size was determined by availability of suitable tissue at the 11 contributing sites
Data exclusions	Data exclusions were performed after quality control for multiple reasons including low tumour content in tissue specimens, low quality nucleic acids, or poor assay performance.
Replication	As this study deals with patient samples, biological replication was not feasible
Randomization	Samples were assigned to groups based on clinical and genomic features such as the anatomical location of the primary tumour, the presence of a TERT or ATRX mutation, or the presence or absence of metastatic disease
Blinding	No blinding was performed in the study

Reporting for specific materials, systems and methods

We require information from authors about some types of materials, experimental systems and methods used in many studies. Here, indicate whether each material, system or method listed is relevant to your study. If you are not sure if a list item applies to your research, read the appropriate section before selecting a response.

Materials & experimental systems

n/a	Involved in the study	
☐	☒	Antibodies
☒	☐	Eukaryotic cell lines
☒	☐	Palaeontology and archaeology
☒	☐	Animals and other organisms
☐	☒	Clinical data
☒	☐	Dual use research of concern
☒	☐	Plants

Methods

n/a	Involved in the study	
☒	☐	ChIP-seq
☒	☐	Flow cytometry
☒	☐	MRI-based neuroimaging

Antibodies

Antibodies used	SDHB (ABCAM, Cambridge, UK: ab14714, clone 21A11, dilution 1:100)<br>Ki67 (clone M7240 Dako, Carpenteria, CA USA, dilution 1:50)
Validation	Routine clinical application

Clinical data

Policy information about clinical studies
All manuscripts should comply with the ICMJEguidelines for publication of clinical research and a completedCONSORT checklist must be included with all submissions.

Clinical trial registration	Not applicable
Study protocol	Not applicable
Data collection	Not applicable
Outcomes	Not applicable

Plants

Seed stocks	Not applicable
Novel plant genotypes	Not applicable
Authentication	Not applicable
